# Efficacy and safety of neoadjuvant chemoimmunotherapy and chemotherapy in patients with potentially resectable stage IIIA/IIIB NSCLC: a retrospective study

**DOI:** 10.3389/fimmu.2024.1479263

**Published:** 2025-01-17

**Authors:** Yuchen Wang, Xiaobo Ma, Kewei Ma, Xi Chen, Hua He, Xiangye Zhao, Mengge Fan, Yinghui Xu

**Affiliations:** ^1^ Cancer Center, The First Hospital of Jilin University, Changchun, Jilin, China; ^2^ Pathological Department, The First Hospital of Jilin University, Changchun, Jilin, China

**Keywords:** non-small cell lung cancer, neoadjuvant therapy, chemoimmunotherapy, potentially resectable, adverse events

## Abstract

**Background:**

Treatment of locally advanced unresectable non-small cell lung cancer (NSCLC) is a significant challenge, especially for patients with IIIA/IIIB NSCLC. Patients receiving neoadjuvant chemoimmunotherapy (NCI) show improved pathological responses and disease-free survival (DFS) compared to those receiving Neoadjuvant chemotherapy (NC). However, there is still no consensus on the treatment for potentially resectable stage IIIA/IIIB NSCLC.

**Methods:**

This retrospective study included 71 patients newly diagnosed with stage III NSCLC at our institution between 2017 and 2023: 46 patients received NCI and 25 patients received NC followed by surgical resection. Their clinicopathological characteristics were reviewed and analyzed.

**Results:**

Patients who received NCI had a significantly longer DFS. The median DFS was 15 months in the NC group (hazard ratio: 0.186, 95% confidence interval[CI]: 0.073–0.479; *P*<0.001) but had not been reached in the NCI group. The percentage of patients achieving a major pathologic response was 65.9% (29/44, 95% CI: 50.0%–79.1%) with NCI and 16.7% (4/24, 95% CI: 5.5%–38.2%) with NC alone(*P*<0.001). The percentage of patients with pathologic complete response was 36.4% (16/44, 95% CI: 22.8%–52.3%) after NCI compared with 8.3% (2/24, 95% CI: 1.5%–28.5%) after NC (*P* = 0.012). The survival curve shows that the overall survival for the NCI group has a better trend than that of the NC group, but there is no significant difference (*P*=0.193). The incidence of all-grade adverse events was greater in the NCI group than in the NC group (80.4% vs. 64.0%). The incidence of grade ≥3 adverse events was 10.9% (n=5) and 8.0% (n=2), respectively; however, these differences were insignificant.

**Conclusions:**

NCI is more effective and safer for patients with potentially resectable stage IIIA/IIIB NSCLC. Compared with NC alone, NCI significantly improves the pathological response and DFS without increasing adverse events.

## 1 Introduction

Lung cancer ranks as the most commonly diagnosed cancer and stands as the leading cause of cancer-related deaths worldwide, accounting for approximately 2.5 million new cases and 1.8 million deaths annually (1), with non-small cell lung cancer (NSCLC) constituting approximately 84% of all new lung cancer diagnoses (2). Despite recent improvements in diagnosis, 30% of patients with NSCLC are diagnosed at stage III (locally advanced disease) (3). Due to the high heterogeneity of locally advanced stage III lung cancers, multidisciplinary teams manage the appropriate curative treatment. Surgical resection is the primary treatment for patients with early-stage NSCLC; the 5-year disease-free survival (DFS) rate is approximately 40% for patients with stage II or III NSCLC. After NSCLC recurrence, the 5-year overall survival (OS) rate is 2–13% (4).

Surgical options are not always available for patients with locally advanced NSCLC. Neoadjuvant treatment followed by surgery is the standard of care for potentially resectable tumors. Neoadjuvant treatment has several advantages, including improved pre-surgical tolerance, tumor downstaging, an earlier opportunity to eradicate micrometastases, improved R0 resection rate, and increased knowledge of drug sensitivity (5, 6). Neoadjuvant chemotherapy (NC) is more beneficial for survival than surgery alone (hazard ratio [HR]: 0.87, 95% confidence interval [CI]: 0.78–0.96; *P*=0.007), reducing the relative mortality risk by 13%, and improving 5-year survival from 40% to 45% (7).

Immune checkpoint inhibitors (ICIs) are immunotherapy-based drugs that improve survival in advanced NSCLC (8). Immune checkpoints are molecules of co-inhibitory signaling pathways that maintain immune tolerance; cancer cells often utilize them to evade immunosurveillance (9). ICIs relieve repression of antitumor immune responses by altering the interactions between T cells and either antigen-presenting cells or tumor cells (10). The most widely used ICI targets are programmed cell death receptor-1 (PD-1), its ligand PD-L1, and cytotoxic T lymphocyte-associated molecule-4 (CTLA-4). Immunotherapy has recently revolutionized the treatment of NSCLC from late to early stages, becoming an integral part of lung cancer treatment.

In addition to providing survival benefits for patients with advanced disease, ICIs can also treat relatively early-stage diseases. The PACIFIC trial studied durvalumab, a PD-L1 inhibitor, in patients with stage III unresectable NSCLC after chemoradiotherapy (11). Patients who received durvalumab showed improved progression-free survival (PFS) compared with placebo (16.8 vs. 5.6 months). This randomized clinical trial (RCT) established a new standard treatment for locally advanced NSCLC. As a consolidation therapy in the GEMSTONE-301 study, sugemalimab after concurrent or sequential chemoradiotherapy significantly lengthened the median PFS compared with placebo (9.0 vs. 5.8 months; *P*=0.0026) in Chinese patients with unresectable stage III NSCLC (12).

Neoadjuvant chemoimmunotherapy (NCI) is increasingly being studied to identify better treatment options. The NADIM study included patients with resectable stage IIIA NSCLC who received three pre-surgical cycles of nivolumab, carboplatin, and paclitaxel and were administered nivolumab post-surgically for one year (13). Among the 46 involved patients, 41 (89%) underwent surgery. The 2-year PFS in patients receiving induction therapy was 77% (95% CI: 60–88). The major pathological response (MPR) rate was 83% (95% CI: 68–93), including a 63% (95% CI: 62–91) pathological complete response (pCR) rate. In the CheckMate 816 randomized trial, 358 patients with stage IB–IIIA NSCLC with wild-type *EGFR* and *ALK* received pre-surgical chemotherapy plus immunotherapy or chemotherapy alone (14). Chemotherapy plus immunotherapy enabled more people to have the surgery (83%) and improved the pCR rate (24% vs. 2%; *P*<0.0001). The LCMC3 trial found a 21% MPR rate after two cycles of neoadjuvant atezolizumab, with an 85% DFS rate at one year in IB–IIIB NSCLC (15).

We focus on patients with locally advanced, potentially resectable, stage IIIA/IIIB NSCLC. We are attempting to apply NCI to transfer these patients from inoperable to operable, enabling advanced patients to achieve long-term survival and a potential clinical cure. A single-arm phase 2 trial enrolled 30 patients with potentially resectable stage IIIA/IIIB NSCLC who underwent NCI for 2–3 cycles; 20 underwent surgical resection (16). The MPR rate was 65% (95% CI: 43.3–82.9%), and the pCR rate reached 40% (95% CI: 21.2–46.3%) in patients who underwent surgery. The 2-year DFS rate in the surgical group stood at 75% (95% CI: 56–94%). Another RCT analyzed patients with stage III resectable NSCLC who received toripalimab or placebo combined with chemotherapy for three cycles pre-surgically and one cycle post-surgically (4). The toripalimab group showed better event-free survival (EFS) (HR: 0.40, 95% CI: 0.28–0.57), MPR rate (48.5% vs. 8.4%), and pCR rate (24.8% vs. 1.0%).

Chemoimmunotherapy is effective as a novel approach to neoadjuvant therapy. However, data are limited for patients with potentially resectable stage III NSCLC who received NCI, particularly regarding long-term follow-up and benefits compared with NC. This study retrospectively analyzed single-center data to compare the effectiveness and safety of NCI and NC in patients with locally advanced NSCLC and determine whether adding ICIs improves long-term outcomes compared with NC alone.

## 2 Methods

### 2.1 Study design

This single-center retrospective study reviewed patients with stage III NSCLC (according to the staging criteria of the American Joint Committee on Cancer, 8th edition) who underwent curative intent surgery after neoadjuvant therapy at The First Bethune Hospital of Jilin University in Changchun, China, between January 2017 and July 2023. Eligible patients were >18 years of age and underwent radical lung cancer surgery with curative intent after platinum-based doublet chemotherapy with or without neoadjuvant ICIs. All patients were treatment-naive. The NCI group’s clinicopathological characteristics and survival data were compared with those of the NC group. Patients with *EGFR* and *ALK* mutations were excluded from the study.

The study adhered to the principles outlined in the Declaration of Helsinki. Written or oral informed consent was obtained from all patients.

### 2.2 Data collection

Baseline demographic and clinical information, such as age, sex, smoking status, histological subtype, and clinical stage, were gathered from medical records. The data were evaluated using imaging information before neoadjuvant treatment, including computed tomography (CT), positron emission tomography plus contrast-enhanced CT (PET-CT), bone scintigraphy, abdominal ultrasound, and brain magnetic resonance imaging. Information was also gathered for neoadjuvant treatments and surgical procedures, including the treatment plan, number of cycles, surgical approach, extent of resection, surgical margins, and pathological stage. Adverse events were evaluated using the National Cancer Institute Common Terminology Criteria for Adverse Events version 5.0.

### 2.3 Outcome evaluation

The study’s primary endpoints were the DFS and MPR rates. DFS was defined as the time from surgery to disease progression or death. The time of progression was defined as the imaging date. MPR was defined as ≤10% viable residual tumor cells identified in the resected specimen. The secondary endpoints were OS, defined as the time from surgery until death, and the pCR rate, characterized by the absence of viable tumor cells in the resected specimen. A multidisciplinary team assessed all cases using the Response Evaluation Criteria in Solid Tumors (RECIST) version 1.1. The outcome categories were complete response (CR), partial response (PR), stable disease (SD), and disease progression (PD). Nodal and tumor downstaging was defined as the change between the clinical N or T stage at the first visit and the ypN or ypT stage after surgery.

### 2.4 Statistical analysis

Statistical analysis was performed using SPSS Statistics version 27.0 (IBM SPSS, IBM Corp.; Armonk, NY, USA). The Fisher’s exact test was used to compare categorical variables. The ordinal continuous variables were assessed using the Wilcoxon rank-sum test. The median follow-up duration was determined through the reverse Kaplan–Meier method. DFS and OS were analyzed through the Kaplan–Meier method and compared using the log-rank test. Univariate and multivariate Cox regression analyses were used to calculate HRs for DFS and OS and to explore prognostic clinical factors. Logistic regression was used for the univariate and multivariate analyses of MPR and pCR. Statistical significance was set at *P*<0.05.

## 3 Results

### 3.1 Patient characteristics

This study enrolled 71 patients diagnosed with stage III NSCLC who underwent surgery between January 2017 and July 2023, divided into NCI (n=46) and NC groups (n=25). The median follow-up time for all patients was 35 months. The baseline demographic and clinical characteristics are summarized in **Table 1**. No significant differences were found between the two groups for sex, age, Eastern Cooperative Oncology Group (ECOG) status, smoking status, comorbidity, histological type, clinical N stage, clinical TNM stage, neoadjuvant cycles, surgical approach, type of resection, or if adjuvant therapy was used (*P*>0.05). The percentage of R0 resections was significantly greater in the NCI group than in the NC group (*P*=0.005). The baseline features of patients in the two treatment groups were balanced.

**Table 1:** Baseline demographic characteristics.

Characteristic	NCI (n=46)	NC (n=25)	*P* value
Sex, n (%)			0.760
Male	38 (82.6)	20 (80.0)	
Female	8 (17.4)	5 (20.0)	
Age, n (%)			0.522
Median (IQR)	58.5 (52.0-64.0)	59 (54.5-64.5)	
≥65	7 (15.2)	6 (24.0)	
<65	39 ( 84.8)	19 (76.0)	
ECOG, n (%)			0.040
0-1	46 (100.0)	22 (88.0)	
2	0 (0.0)	3 (12.0)	
Smoking status, n (%)			1.000
Former/current-smoker	1 8(39.1)	10 (40.0)	
Non-smoker	28 (60.9)	15 (60.0)	
Comorbidities, n (%)			1.000
Yes	15 (32.6)	8 (32.0)	
No	31 (67.4)	17 (68.0)	
Histological Type, n (%)			0.522
Squamous cell	39 (84.8)	19 (76.0)	
Adenocarcinoma	7 (15.2)	6 (24.0)	
cT stage, n (%)			0.747
T1	7 (15.2)	2 (8.0)	
T2	8 (17.4)	5 (20.0)	
T3	7 (15.2)	8 (32.0)	
T4	24 (52.2)	10 (40.0)	
cN stage, n (%)			0.905
N0	2 (4.4)	5 (20.0)	
N1	18 (39.1)	4 (16.0)	
N2	23 (50.0)	15 (60.0)	
N3	3 (6.5)	1 (4.0)	
cTNM stage, n (%)			0.445
IIIA	33 (71.7)	16 (64.0)	
IIIB	13 (28.3)	8 (32.0)	
IIIC	0 (0.0)	1 (4.0)	
Neoadjuvant cycles, n (%)			0.453
≤2	24 (52.2)	16 (64.0)	
>2	22 (47.8)	9 (36.0)	
Surgical approach, n (%)			0.337
Minimally invasive surgery	44 (95.7)	22 (88.0)	
Open thoracotomy	2 (4.3)	3 (12.0)	
Type of resection, n (%)			0.520
Lobectomy	37 (80.4)	22 (88.0)	
Pneumonectomy	9 (19.6)	3 (12.0)	
Surgical margin, n (%)			0.004
R0	44 (95.7)	19 (76.0)	
R1	0 (0.0)	5 (20.0)	
Unknown	2 (4.3)	1 (4.0)	
Adjuvant therapy, n (%)			0.439
Yes	40 (87.0)	20 (80.0)	
No	6 (13.)	5 (20.0)	

NCI, neoadjuvant chemoimmunotherapy; NC, neoadjuvant chemotherapy; ECOG, Eastern Cooperative Oncology Group; cT stage, clinical T stage before treatment; cN stage, clinical N stage before treatment; cTNM stage, clinical TNM stage before treatment.

### 3.2 Pathological and radiological assessment

**Figure 1:**
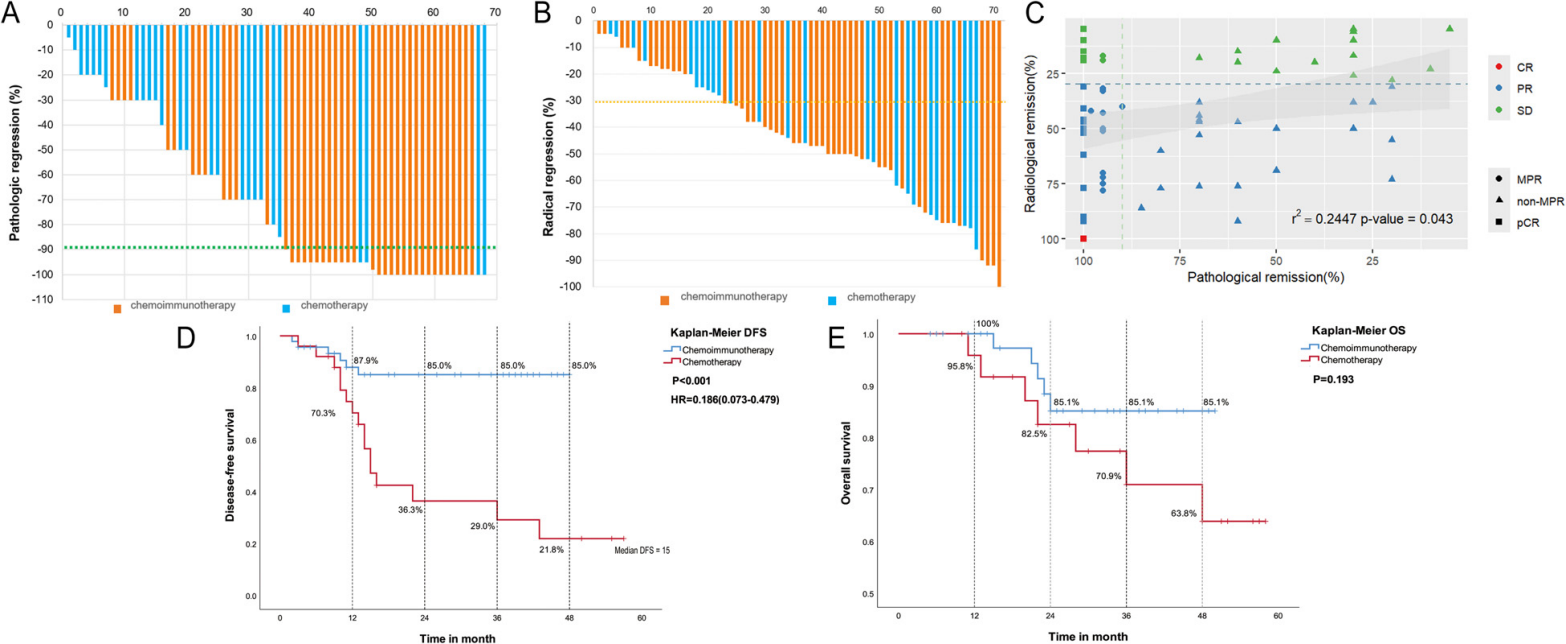
Analysis of efficacy. **(A)** Waterfall plot of pathological response in two groups; **(B)** Waterfall plot of radiological response in the neoadjuvant chemoimmunotherapy group and neoadjuvant chemotherapy group; **(C)** Correlation between pathological remission and radiological remission; **(D)** Disease-free survival (DFS) according to different neoadjuvant therapy types. **(E)** Overall survival (OS) according to different neoadjuvant therapy types.

We retrospectively analyzed the clinical and pathological data of the two groups of patients and compared their efficacies (**Table 2**; **Figure 1A**). Pathological response data were obtained for 68 patients (44 NCI and 24 NC). Data from 3 cases were unavailable because the operations of these patients were not in our center. In the NCI group, 35/46 patients (76.1%) achieved pathological tumor downstaging, and 27/46 patients (58.7%) achieved pathological nodal downstaging in the NCI group compared to 18/25 patients (72.0%) and 10/25 patients (40.0%) in the NC group, with no significant difference (*P*>0.05). However, 29/44 patients (65.9%, 95% CI: 50.0%–79.1%) achieved MPR, and 16 (36.4%, 95% CI: 22.8%–52.3%) achieved pCR, significantly greater than the MPR (4/24, 16.0%, 95% CI: 5.5%–38.2%; *P*<0.001) and pCR (2/24, 8.0%, 95% CI: 1.5%–28.5%; *P*=0.012) rates in the NC group, respectively).

**Table 2:** Pathological and radiological response in NSCLC population.

Characteristic	NCI (n=46)	NC (n=25)	χ^2^/Z value	*P* value
Tumor downstaging, n (%)^a^			0.187	0.666
Yes	35 (76.1)	18 (72.0)		
No	9 (19.6)	6 (24.0)		
Nodal downstaging, n (%)^a^			2.429	0.119
Yes	27 (58.7)	10 (40.0)		
No	17 (40.0)	14 (56.0)		
Major pathological response, n (%)^a^			15.075	<0.001
Yes	29 (65.9)	4 (16.7)		
No	15 (34.1)	20 (83.3)		
Pathological complete response, n (%)^a^			6.269	0.012
Yes	16 (34.8)	2 (8.3)		
No	28 (60.9)	22 (91.6)		
Radiological response, n (%)			1.295	0.195
CR	1 (2.2)	0 (0.0)		
PR	33 (71.7)	15 (60.0)		
SD	12 (26.1)	10 (40.0)		

a In three cases data were not available.

NCI, neoadjuvant chemoimmunotherapy; NC, neoadjuvant chemotherapy; CR, complete response; PR, partial response; SD, stable disease.

According to the RECIST1.1 criteria, one patient in the NCI group achieved CR, 33 achieved PR, and 12 had SD (**Table 2**; **Figure 1B**). In the NC group, 15 (60.0%) achieved a PR, 10 (40.0%) maintained SD, and no patient achieved CR. The NCI group shows a greater objective response rate (34/46, 73.9%, 95% CI: 61.2–86.6%) than the NC group (15/25, 60.0%, 95% CI: 40.8–79.2%) (P=0.123). No patients achieved PD in either group; the disease control rate (DCR) was 100.0%. Spearman’s correlation analysis showed that the pathological remission rate is positively correlated with the radiological remission rate (R=0.2447; P=0.043) (**Figure 1C**).

We performed univariate and multivariate logistic regression analyses of MPR and pCR in 71 patients with NSCLC treated with NC or NCI. The neoadjuvant therapy type was identified as an independent factor affecting MPR (HR=11.442, 95% CI: 2.982–43.898; P<0.001) and pCR (HR=7.215, 95% CI: 1.436–36.256; P=0.016) (**Table 3**).

**Table 3:** Logistic analysis of pathological response for 71 NSCLC patients treated with neoadjuvant chemotherapy or chemoimmunotherapy.

Variate	MPR				pCR			
	Univariate		Multivariate		Univariate		Multivariate	
	OR(95%CI)	*P* value	OR(95%CI)	*P* value	OR(95%CI)	*P* value	OR(95%CI)	*P* value
Neoadjuvant therapy type		<0.001		<0.001^a^		0.022		0.016^b^
Chemotherapy	1		1		1		1	
Chemoimmunotherapy	9.667 (2.794-33.450)		11.442 (2.982-43.898)		6.286 (1.305-30.288)		7.215 (1.436-36.256)	

MPR, major pathological response; pCR, pathological complete response.

a Adjusted for comorbidities.

b Adjusted for smoking status.

### 3.3 Survival outcomes and prognostic factors

As of 31 December 2023, 6/46 patients (13%) in the NCI group had a postoperative recurrence, with a median DFS not reached; 16/25 patients (64%) in the NC group showed recurrence, with a median DFS of 15 months. The NCI group showed a significant survival advantage over the NC group (median DFS not reached vs. 15 months: HR=0.186, 95% CI: 0.073–0.479; *P*<0.001). The 1-, 2-, 3-, and 4-year DFS rates in the NCI group were superior to those in the NC group: 87.9% (95%CI: 78.5%–97.3%) vs. 70.3% (95%CI: 52.4%–88.2%), 85.0% (95%CI: 74.7%–95.3%) vs. 36.3% (95%CI: 17.5%–55.1%), 85.0% (95%CI: 74.7%–95.3%) vs. 29.0% (95%CI: 52.4%–88.2%), and 85.0% (95%CI: 74.7%–95.3%) vs. 21.8% (95%CI: 5.6%–38.0%), respectively (**Figure 1D**).

By the follow-up date, deaths had occurred in the NCI (n=5) and NC (n=7) groups; the median OS was not reached in either group. Survival curves showed better long-term survival in the NCI group than in the NC group; however, the difference was insignificant (*P*=0.193). The 1-, 2-, 3-, and 4-year OS rates were greater in the NCI group than in the NC group: 100.0% vs. 95.8%, 85.1%(95%CI: 74.8%–95.4%) vs. 82.5%(95%CI: 67.6%–97.4%), 85.1%(95%CI:74.8%–95.4%) vs. 70.9%(95%CI: 53.1%–88.7%), and 85.1%(95%CI: 74.8%–95.4%) vs. 63.8%(95%CI: 45.0%–82.6%), respectively (**Figure 1E**).

The baseline characteristics of the entire population were included in the univariate Cox regression model to explore their effect on the relationship between different treatment modalities and survival outcomes. The Cox multivariate analysis included influencing factors with *P*<0.05 in the univariate analysis. As shown in **Table 4**, the neoadjuvant therapy type was an independent prognostic factor for DFS (HR=0.242, 95% CI: 0.089–0.658; P<0.001). In the univariate analysis, DFS was associated with MPR (*P*=0.016), pCR (*P*=0.023), and nodal downstaging (*P*=0.011) (**Supplementary Figure 1**). However, multivariate Cox regression analysis showed that the neoadjuvant therapy type might not be an independent prognostic factor for OS (*P*=0.817).

**Table 4:** Comparison of different neoadjuvant therapy types in univariate and multivariate Cox regression analyzes for survival.

Variate	DFS				OS			
	Univariate		Multivariate		Univariate		Multivariate	
	HR(95%CI)	*P* value	HR(95%CI)	*P* value	HR(95%CI)	*P* value	HR(95%CI)	*P* value
Neoadjuvant therapy type		<0.001		0.005^a^		0.206		0.817^b^
Chemotherapy	1		1		1		1	
Chemoimmunotherapy	0.186 (0.073-0.479)		0.242 (0.089-0.658)		0.473 (0.148-1.508)		0.853 (0.222-3.273)	

DFS, disease-free survival; OS, overall survival.

a Adjusted for surgical margin.

b Adjusted for surgical margin and cT stage.

### 3.4 Subgroup analysis for survival

We performed subgroup analyses of DFS, OS, MPR, and pCR to compare the efficiency of different neoadjuvant therapies in patients with the same clinical characteristics. The HR for DFS was <1.00 in most subgroups, indicating that the NCI group had a better prognosis than the NC group for most subgroups. Patients in the <65 age, ECOG score 0–1, stage IIIA, and N2-positive subgroups benefitted from NCI, showing significant improvement in DFS compared with the NC group (*P*<0.05) (**Supplementary Table 1**; **Supplementary Figure 2A**). In the majority of subgroups, The HR for OS was <1.00, however, the difference was insignificant (**Supplementary Table 1**; **Supplementary Figure 2B**). In addition, most subgroups’ MPR and pCR rates favored NCI over NC, with an HR>1.00 (**Supplementary Table 2**; **Supplementary Figures 2C, D**).

Subgroup analyses based on the clinical characteristics of the NCI group were performed to investigate possible populations (**Table 5**). The analysis revealed significantly better DFS in male than in female patients (*P*=0.019); however, no significant differences were found in OS, MPR, or pCR (*P >*0.05). Patients with squamous cell lung cancer were more likely to achieve pCR than those with adenocarcinoma (*P*=0.037). NCI in patients with stage IIIA NSCLC resulted in insignificantly better OS (*P*=0.056). In the subgroup analysis of age (<65 vs. ≥65 years), ECOG, histological type (adenocarcinoma vs. squamous cell), cTNM stage (IIIA vs. IIIB), and treatment cycles (≤2 cycles vs. >2 cycles), no significant differences were found between the clinical characteristics (all *P*>0.05).

**Table 5:** Subgroup analysis of DFS, OS, MPR, and pCR by clinical characteristics in the neoadjuvant immunotherapy group.

Variate	DFS		OS		MPR		pCR	
	*P* value	HR(95%CI)	*P* value	HR(95%CI)	*P* value	OR(95%CI)	P value	OR(95%CI)
Sex (Male/Female)	**0.019**	0.182 (0.037-0.904)	0.829	0.786 (0.088-7.037)	0.695	0.596 (0.052-4.005)	1.000	0.943 (0.153-7.069)
Age (≥65/<65)	0.921	1.115 (0.130-9.544)	0.118	3.777 (0.630-22.650)	0.395	0.470 (0.055-4.038)	1.000	0.860 (0.069-6.932)
ECOG(0/1)	0.099	–	0.136	–	1.000	1.232 (0.262-6.778)	0.314	0.423 (0.062-2.107)
Smoking status (Former or current-smoker/Non-smoker)	0.193	2.930 (0.536-16.010)	0.770	0.766 (0.128-4.589)	0.521	0.609 (0.141-2.607)	0.057	0.239 (0.036-1.142)
Histological Type (Adenocarcinoma/Squamous cell)	0.983	1.024 (0.119-8.788)	0.335	–	0.207	0.327 (0.041-2.282)	**0.037**	0.000 (0.000-1.085)
cN stage (N0,N1/N2)	0.641	0.745 (0.136-4.076)	0.393	0.423 (0.047-3.785)	0.744	1.423 (0.327-6.503)	0.058	3.774 (0.868-18.783)

P-values less than 0.05 are marked in bold.

### 3.5 Comparison of treatment after disease recurrence

We analyzed treatment options for patients with disease recurrence (**Supplementary Figure 3**). In the NCI group, disease recurred in 6 patients (13.0%). Three of these patients were treated with chemoimmunotherapy or immunotherapy alone; radiotherapy was administered to one patient. Sixteen patients (64.0%) had disease recurrence in the NC group; 4 were treated with chemoimmunotherapy or immunotherapy alone after relapse, 6 with chemotherapy, and 6 with radiotherapy.

### 3.6 Safety analysis

During neoadjuvant therapy, the groups showed different degrees of treatment-related adverse events (TRAEs); no treatment-related deaths occurred. The total incidence of adverse reactions was 80.4% in the NCI group and 64.0% in the NC group; the rates of grade ≥3 adverse reactions were 10.9% (n=5) and 8.0% (n=2), respectively, with no significant difference (*P*=0.128, *P*=0.662), suggesting that the safety of the two regimens was similar and acceptable (**Table 6**). The three most common adverse reactions were leukopenia, neutropenia, and anemia. The NCI group’s most common grade ≥3 TRAE was neutropenia (n=4, 8.7%). The most common adverse event of grade ≥3 in the NC group was neutropenia (n=4, 8.7%), followed by leukopenia (n=1, 2.2%).

**Table 6:** Comparison of adverse events in two groups.

Class of adverse events	NCI (n=46)		NC (n=25)	
	Any grade (N,%)	Grade3-4 (N,%)	Any grade (N, %)	Grade3-4 (N,%)
Blood and lymphatic system disorders				
Leukopenia	11 (23.9)	1 (2.2)	5 (20.0)	0
Neutropenia	12 (26.1)	4 (8.7)	7 (28.0)	1 (4.0)
Thrombocytopenia	4 (13.0)	0	2 (8.0)	1 (4.0)
Anemia	13 (28.3)	0	6 (24.0)	0
Hepatobiliary disorders				
Aminotransferase increased	10 (21.7)	0	0	0
Gamma glutamyl transferase increased	7 (15.2)	0	1 (4.0)	0
Hypoalbuminemia	11 (23.9)	0	3 (12.0)	0
Renal and urinary disorders				
Proteinuria	1 (2.2)	0	0	0
Creatinine increased	0	0	1 (4.0)	0
Gastrointestinal disorders				
Nausea	3 (6.5)	0	2 (8.0)	0
Vomiting	2 (4.3)	0	0	0
Abdominal distension	0	0	1 (4.0)	0
Constipation	1 (2.2)	0	0	0
Gastrointestinal ulcer	0	0	1 (4.0)	0
Respiratory disorders				
Dyspnea	0	0	1 (4.0)	0
Endocrine disorders				
Hypothyroidism	1 (2.2)	0	0	0
General disorders				
Fever	2 (4.3)	0	1 (4.0)	0
Dizziness	0	0	1 (4.0)	0
Malaise	1 (2.2)	0	1 (4.0)	0
Arthralgia	0	0	1 (4.0)	0
Skin and subcutaneous tissue disorders				
Rash	0	0	1 (4.0)	0

NCI, neoadjuvant chemoimmunotherapy; NC, neoadjuvant chemotherapy.

## 4 Discussion

This retrospective clinical study analyzed 71 patients with locally advanced stage IIIA/IIIB NSCLC treated with curative-intent surgery after NCI or NC alone. Our analysis suggests that neoadjuvant therapy converts unresectable tumors into resectable tumors. In addition, the NCI group showed better efficacy and long-term survival than the NC group.

The PACIFIC trial, which significantly influenced clinical practice, established concurrent chemoradiotherapy followed by consolidation immunotherapy as the standard treatment for patients with unresectable stage III NSCLC (11). Moreover, NCI substantially improves the pathological response in resectable IB–IIIA NSCLC (13, 17, 18). NCI has the potential to downstage initially unresectable NSCLC to a resectable state, markedly enhancing survival outcomes in patients undergoing surgery compared to those who do not; however, this finding should be validated in RCTs. Given that RCTs are time-consuming, expensive, and retard progress in locally advanced NSCLC, we conducted this retrospective study to evaluate whether NCI effectively converts NSCLC from unresectable to resectable and collect more data on DFS and OS.

In this study, the MPR rate was significantly greater in the NCI group than in the NC group. Patients with MPR had significantly better DFS than those with incomplete pathological responses, confirming that combining immunotherapy with NC improves the pathological response. A meta-analysis of 53 clinical trials reported that MPR is associated with improved OS (HR=0.80, 95% CI: 0.72–0.88; *P*<0.001) compared with non-MPR and significantly enhanced DFS/PFS/EFS (HR=0.28, 95% CI: 0.10–0.79; *P*=0.02) (19), suggesting MPR prolongs OS and DFS. The combined effect of chemotherapy and ICIs may explain the greater MPR rate, with cytotoxic chemotherapy enhancing the immunotherapeutic effects of ICIs (20).

In addition, the DFS was significantly better in the NCI groups than the NC groups in our study, confirming that the NCI regimen is an independent prognostic factor for DFS. Our analysis suggests that patients experience a more durable survival benefit from combination immunochemotherapy. Although the NCI group showed a better survival curve than the NC group, no significant difference was found. However, the NCI group’s 1-, 2-, 3- and 4‐year OS rates were greater than those of the NC group, suggesting that patients may benefit from NCI. Therefore, NCI might improve disease outcomes compared to NC alone in potentially resectable stage IIIA/IIIB NSCLC. It may also show better efficacy for pathological remission and, thus, a better prognosis.

The DFS benefit did not translate into significantly improved OS, possibly because of a crossover effect of subsequent therapies. Some patients in the NC group received additional immunotherapy after relapse, and some patients in the NCI group discontinued immunity after relapse, resulting in no significant difference in OS between the NCI group and NC group. One study that analyzed 1201 patients with NSCLC treated with PD-(L)1 blockade found that acquired resistance occurred in >60% of initial responders (21). This result suggests that acquired resistance might lessen the DFS’s effect on OS. In addition, our sample size was small, possibly biasing the analysis. The OS results should be validated in larger RCTs.

Precisely identifying the population suitable for NCI is essential. Our analysis showed that male patients benefitted more from NCI (*P*=0.019). Tuminello et al. reported that among patients with squamous cell histology, males derived more survival benefits from chemoimmunotherapy than female patients (*P*=0.07); the percentage of squamous cells was significantly greater in male patients (87.9% vs. 53.8%; *P*=0.004), possibly explaining this result. Moreover, no significant differences were found in DFS, OS, MPR, or pCR between ≤2 and >2 treatment cycles, suggesting that increasing the therapy cycles may not improve efficacy. One observational two-arm clinical study (22) found a greater MPR rate after three therapy cycles than with two. However, a retrospective study found that patients receiving two therapy cycles had significantly smaller diagnostic radiological tumor size (37.0 mm vs. 49.6 mm; *P*=0.022) than patients receiving >2 cycles but no significant difference in the pathological tumor regression rate (23).

Safety must be considered in NCI. The NADIM trial, a clinical trial of NCI for NSCLC, reported 93% (43/46) of patients had TRAEs during neoadjuvant treatment; the most common grade ≥3 TRAEs were increased lipase (n=3, 7%) and febrile neutropenia (n=3, 7%) (13). The Checkmate 816 trial found a 92.6% rate of any grade TRAE in the nivolumab combination chemotherapy group, with a 33.5% incidence of grade 3 or 4 adverse events; neutropenia was the most common (24). Our study’s total TRAE rate in the NCI group (80.4%) was less than that of the NADIM and Checkmate 816 studies. The NCI group’s most common grade ≥3 TRAE was neutropenia (n=4, 8.7%), which healed quickly. Furthermore, NCI did not increase TRAEs compared to NC, confirming its safety. Although studies suggest that the NCI’s safety is generally manageable, whether serious adverse events cause disease progression, surgical delays, or even death needs verification in a significant number of clinical trials.

Apart from what was described above, this study focuses on locally advanced stage IIIA/IIIB NSCLC, for which there is a deficiency in clinical data (**Supplementary Table 3**). Moreover, there is a great difference between clinical practice in the real world and clinical trials. In clinical trials, patients typically adhere to strict experimental protocols, but in daily clinical practice, patients’ preferences, as well as their physical and economic conditions, significantly impact treatment decisions. Therefore, our research aims to compile real-world data to guide future treatment strategies.

Although the real-world study can take into account more of the patients’ physical and economic factors as well as their personal choices, which is more in line with real clinical situations, our study had some limitations. First, this was a single-center retrospective analysis with a small sample size, and the difference in the duration of follow-up and baseline characteristics between retrospective groups may have led to bias. The beneficial trends observed in this study need to be confirmed by more prospective clinical studies. Second, as only patients who underwent surgery was enrolled in this study, it cannot be concluded how many patients with clinical stage IIIA/IIIB disease started neoadjuvant therapy but were not able to proceed with surgery. Third, the long-term survival data were insufficient. The median OS for both groups was not reached, necessitating a longer follow-up period to obtain reliable OS data. Fourth, this study remains in its initial stages. Potential predictive markers of efficacy (PD-L1 expression, lymphocyte subsets, and changes in tumor marker levels) were not included. Long-term RCTs are needed to address these limitations, and further investigation into the molecular mechanisms of NCI is warranted. Despite these limitations, this real-world retrospective study provides an objective analysis of the efficacy, safety, and survival outcomes of NCI in potentially resectable stage IIIA/IIIB NSCLC.

## 5 Conclusions

Compared with NC alone, NCI significantly improves the pathological response and DFS of patients with potentially resectable stage IIIA/IIIB NSCLC without increasing adverse events. In addition, our analysis suggests that NCI treatment can enable more patients to be eligible for resection and improve their long-term survival. However, our findings require verification in a large-scale RCT with a sufficient follow-up period.

## Data Availability

The original contributions presented in the study are included in the article/**Supplementary Material**. Further inquiries can be directed to the corresponding author.
