# Supplementary material for: Efficacy and safety of neoadjuvant chemoimmunotherapy and chemotherapy in patients with potentially resectable stage IIIA/IIIB NSCLC: a retrospective study
Source: Front Immunol. 2025 Jan 17;15:1479263. doi: 10.3389/fimmu.2024.1479263 (PMC11782129; doi:10.3389/fimmu.2024.1479263)
Supplement: Supplementary file 1 [file DataSheet1.docx]

Supplementary Material

# Supplementary Figures


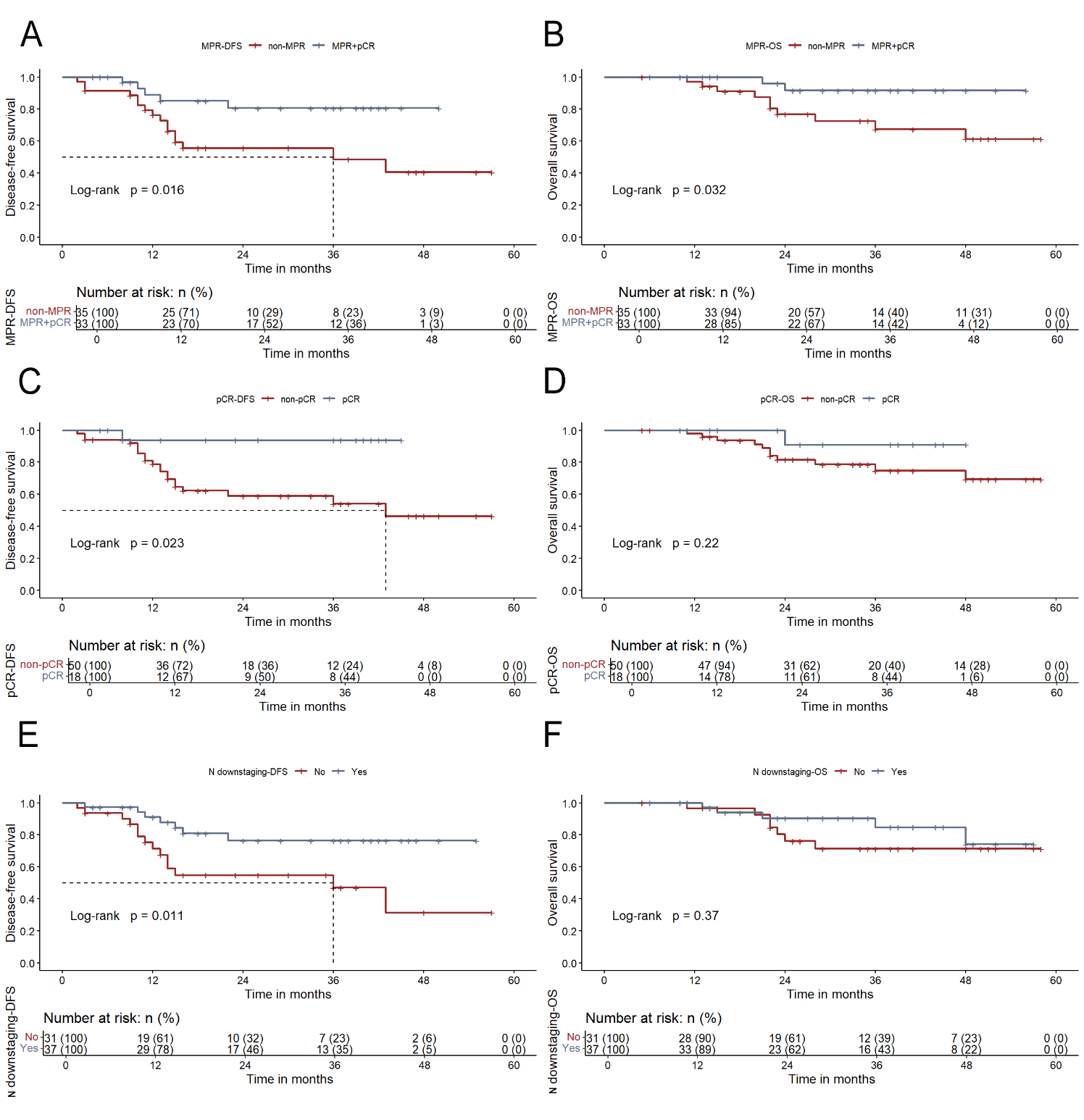


Supplementary Figure 1. A–B, The DFS (A) and OS (B) curves for MPR and non-MPR patients with potentially resectable stage IIIA/B NSCLC who received neoadjuvant chemoimmunotherapy or neoadjuvant chemotherapy; C–D, The DFS (C) and OS (D) curves for pCR and non- pCR patients; E–F, The DFS (E) and OS (F) curves for patients with or without lymph node downstaging. DFS, disease-free survival; OS, overall survival; NSCLC, non-small-cell lung cancer; MPR, major pathological response; pCR, pathological complete response.


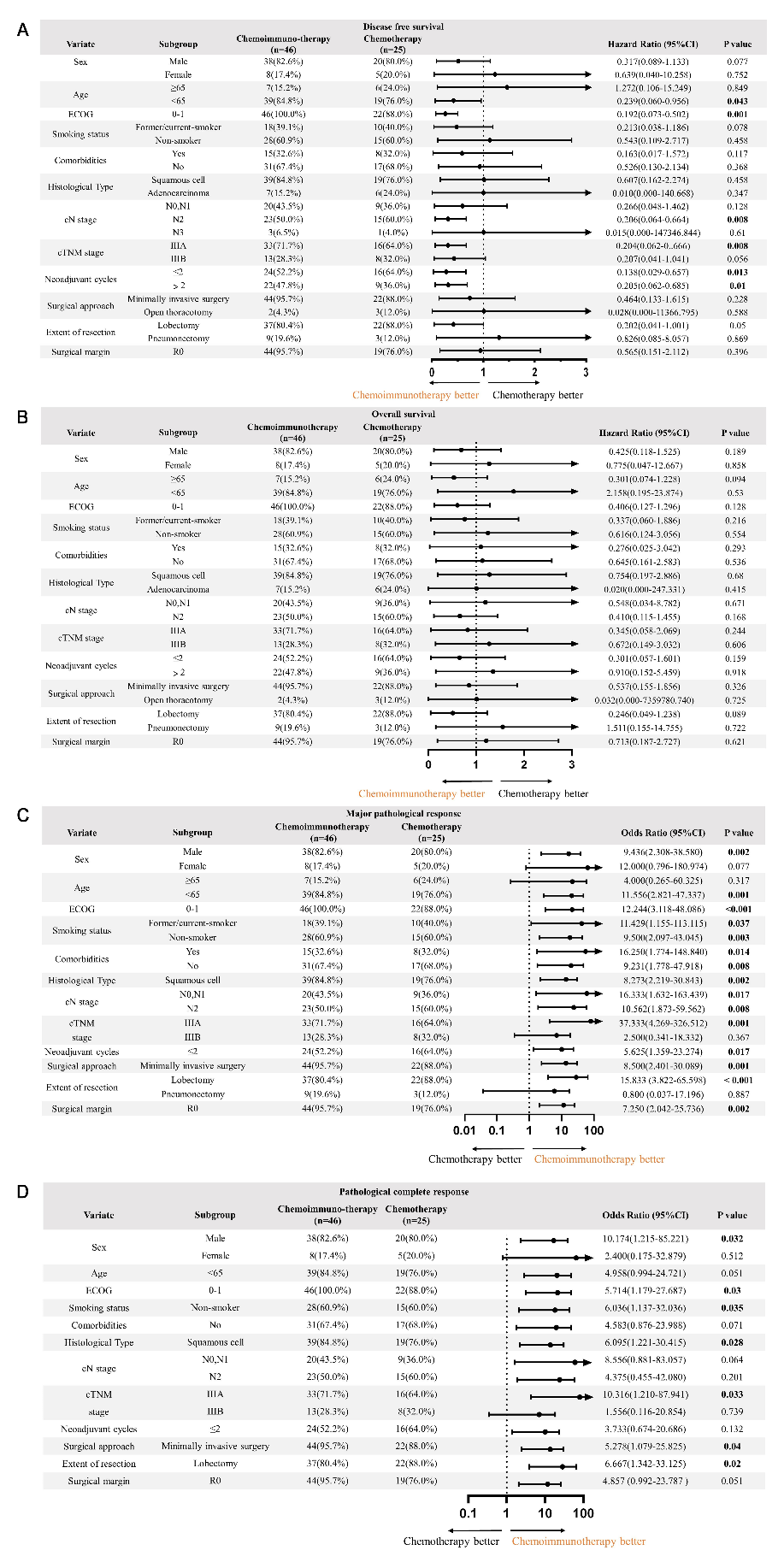


Supplementary Figure 2. Subgroup analysis of disease-free survival (A), overall survival (B), major pathological response (C), pathological complete response (D).


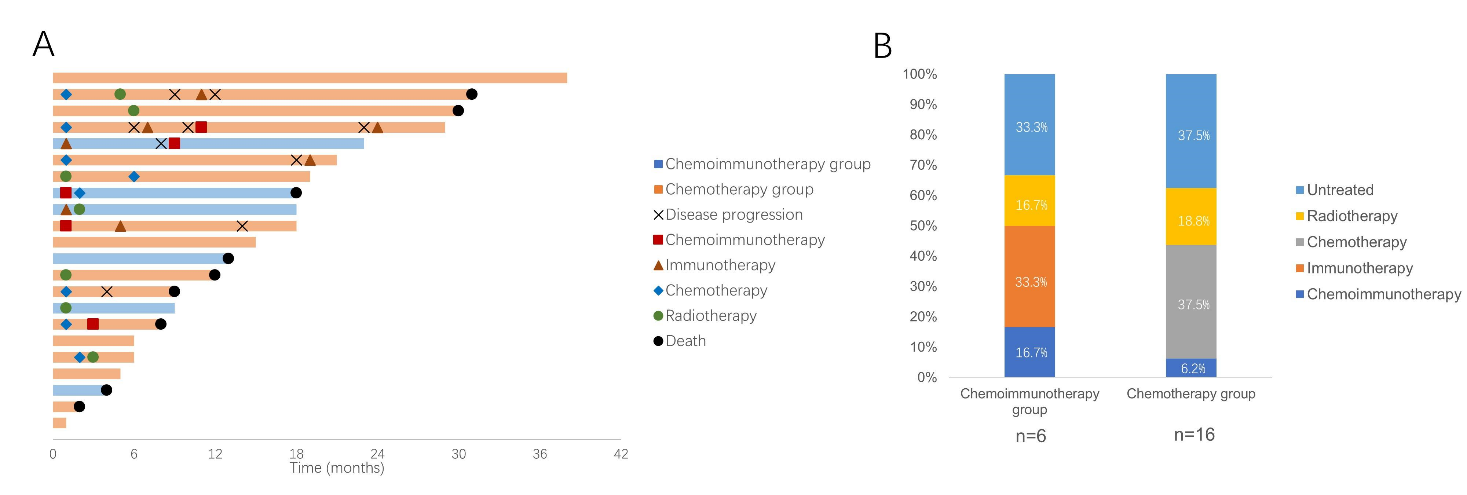


Supplementary Figure 3. Treatment of patients after relapse. (A) Swimming plot, each bar represents one patient. (B) Percentage of the first treatment after relapse.

# Supplementary Tables

**Supplementary Table 1.** Subgroup analysis of prognostic factors for disease-free survival (DFS) and overall survival (OS) in 71 NSCLC patients treated with neoadjuvant chemotherapy or chemoimmunotherapy.

| Variate | Subgroup | Chemoimmunotherapy (n=46) | Chemotherapy  (n=25) | DFS | | OS | |
| --- | --- | --- | --- | --- | --- | --- | --- |
|  |  |  |  | HR (95%CI) | P value | HR (95%CI) | P value |
| Sex | Male | 38(82.6%) | 20(80.0%) | 0.317(0.089-1.133) | 0.073 | 0.425(0.118-1.525) | 0.189 |
|  | Female | 8(17.4%) | 5(20.0%) | 0.639(0.040-10.258) | 0.752 | 0.775(0.047-12.667) | 0.858 |
| Age | ≥65 | 7(15.2%) | 6(24.0%) | 1.272(0.106-15.249) | 0.849 | 0.301(0.074-1.228) | 0.094 |
|  | <65 | 39(84.8%) | 19(76.0%) | 0.239(0.060-0.956) | **0.043** | 2.158(0.195-23.874) | 0.530 |
| ECOG | 0-1 | 46(100.0%) | 22(88.0%) | 0.192(0.073-0.502) | **0.001** | 0.406(0.127-1.296) | 0.128 |
|  | 2 | 0(0.0%) | 3(12.0%) | - | - | - | - |
| Smoking status | Former/current-smoker | 18(39.1%) | 10(40.0%) | 0.213(0.038-1.186) | 0.078 | 0.337(0.060-1.886) | 0.216 |
|  | Non-smoker | 28(60.9%) | 15(60.0%) | 0.543(0.109-2.717) | 0.458 | 0.616(0.124-3.056) | 0.554 |
| Comorbidities | Yes | 15(32.6%) | 8(32.0%) | 0.163(0.017-1.572) | 0.117 | 0.276(0.025-3.042) | 0.293 |
|  | No | 31(67.4%) | 17(68.0%) | 0.526(0.130-2.134) | 0.368 | 0.645(0.161-2.583) | 0.536 |
| Histological Type | Squamous cell | 39(84.8%) | 19(76.0%) | 0.607(0.162-2.274) | 0.458 | 0.754(0.197-2.886) | 0.680 |
|  | Adenocarcinoma | 7(15.2%) | 6(24.0%) | 0.010(0.000-140.668) | 0.347 | 0.020(0.000-247.331) | 0.415 |
| cN stage | N0,N1 | 20(43.5%) | 9(36.0%) | 0.266(0.048-1.462) | 0.128 | 0.548(0.034-8.782) | 0.671 |
|  | N2 | 23(50.0%) | 15(60.0%) | 0.206(0.064-0.664) | **0.008** | 0.410(0.115-1.455) | 0.168 |
|  | N3 | 3(6.5%) | 1(4.0%) | 0.015(0.000-147346.844) | 0.610 | - | - |
| cTNM stage | IIIA | 33(71.7%) | 16(64.0%) | 0.204(0.062-0.666) | **0.008** | 0.345(0.058-2.069) | 0.244 |
|  | IIIB | 13(28.3%) | 8(32.0%) | 0.207(0.041-1.041) | 0.056 | 0.672(0.149-3.032) | 0.606 |
|  | IIIC | 0(0.0%) | 1(4.0%) | - | - | - | - |
| Neoadjuvant cycles | ≤2 | 24(52.2%) | 16(64.0%) | 0.138(0.029-0.657) | **0.013** | 0.301(0.057-1.601) | 0.159 |
|  | ＞2 | 22(47.8%) | 9(36.0%) | 0.205(0.062-0.685) | **0.010** | 0.910(0.152-5.459) | 0.918 |
| Surgical approach | Minimally invasive surgery | 44(95.7%) | 22(88.0%) | 0.464(0.133-1.615) | 0.228 | 0.537(0.155-1.856) | 0.326 |
|  | Open thoracotomy | 2(4.3%) | 3(12.0%) | 0.028(0.000-11366.795) | 0.588 | 0.032(0.000-7359780.740) | 0.725 |
| Extent of resection | Lobectomy | 37(80.4%) | 22(88.0%) | 0.202(0.041-1.001) | 0.050 | 0.246(0.049-1.238) | 0.089 |
|  | Pneumonectomy | 9(19.6%) | 3(12.0%) | 0.826(0.085-8.057) | 0.869 | 1.511(0.155-14.755) | 0.722 |
| Surgical margin | R0 | 44(95.7%) | 19(76.0%) | 0.565(0.151-2.112) | 0.396 | 0.713(0.187-2.727) | 0.621 |
|  | R1 | 0(0.0%) | 5(20.0%) | - | - | - | - |

**Supplementary Table 2.** Subgroup analysis of prognostic factors for major pathological response (MPR) and pathological complete response (pCR) in 71 NSCLC patients treated with neoadjuvant chemotherapy or chemoimmunotherapy.

| Variate | Subgroup | Chemoimmunotherapy (n=46) | Chemotherapy  (n=25) | MPR | | PCR | |
| --- | --- | --- | --- | --- | --- | --- | --- |
|  |  |  |  | OR (95%CI) | P-value | OR (95%CI) | P-value |
| Sex | Male | 38(82.6%) | 20(80.0%) | 9.436(2.308-38.580) | **0.002** | 10.174(1.215-85.221) | **0.032** |
|  | Female | 8(17.4%) | 5(20.0%) | 12.000(0.796-180.974) | 0.077 | 2.400(0.175-32.879) | 0.512 |
| Age | ≥65 | 7(15.2%) | 6(24.0%) | 4.000(0.265-60.325) | 0.317 | - | - |
|  | <65 | 39(84.8%) | 19(76.0%) | 11.556(2.821-47.337) | **0.001** | 4.958(0.994-24.721) | 0.051 |
| ECOG | 0-1 | 46(100.0%) | 22(88.0%) | 12.244(3.118-48.086) | **<0.001** | 5.714(1.179-27.687) | **0.030** |
|  | 2 | 0(0.0%) | 3(12.0%) | - | - | - | - |
| Smoking status | Former/current-smoker | 18(39.1%) | 10(40.0%) | 11.429(1.155-113.115) | **0.037** | - | - |
|  | Non-smoker | 28(60.9%) | 15(60.0%) | 9.500(2.097-43.045) | **0.003** | 6.036(1.137-32.036) | **0.035** |
| Comorbidities | Yes | 15(32.6%) | 8(32.0%) | 16.250(1.774-148.840) | **0.014** | - | - |
|  | No | 31(67.4%) | 17(68.0%) | 9.231(1.778-47.918) | **0.008** | 4.583(0.876-23.988) | 0.071 |
| Histological Type | Squamous cell | 39(84.8%) | 19(76.0%) | 8.273(2.219-30.843) | **0.002** | 6.095(1.221-30.415) | **0.028** |
|  | Adenocarcinoma | 7(15.2%) | 6(24.0%) | - | - | - | - |
| cN stage | N0,N1 | 20(43.5%) | 9(36.0%) | 16.333(1.632-163.439) | **0.017** | 8.556(0.881-83.057) | 0.064 |
|  | N2 | 23(50.0%) | 15(60.0%) | 10.562(1.873-59.562) | **0.008** | 4.375(0.455-42.080) | 0.201 |
|  | N3 | 3(6.5%) | 1(4.0%) | - | - | - | - |
| cTNM  stage | IIIA | 33(71.7%) | 16(64.0%) | 37.333(4.269-326.512) | **0.001** | 10.316(1.210-87.941) | **0.033** |
|  | IIIB | 13(28.3%) | 8(32.0%) | 2.500(0.341-18.332) | 0.367 | 1.556(0.116-20.854) | 0.739 |
|  | IIIC | 0(0.0%) | 1(4.0%) | - | - | - | - |
| Neoadjuvant cycles | ≤2 | 24(52.2%) | 16(64.0%) | 5.625(1.359-23.274) | **0.017** | 3.733(0.674-20.686) | 0.132 |
|  | ＞2 | 22(47.8%) | 9(36.0%) | - | - | - | - |
| Surgical approach | Minimally invasive surgery | 44(95.7%) | 22(88.0%) | 8.500(2.401-30.089) | **0.001** | 5.278(1.079-25.825) | **0.040** |
|  | Open thoracotomy | 2(4.3%) | 3(12.0%) | - | - | - | - |
| Extent of resection | Lobectomy | 37(80.4%) | 22(88.0%) | 15.833 (3.822-65.598) | **< 0.001** | 6.667(1.342-33.125) | **0.020** |
|  | Pneumonectomy | 9(19.6%) | 3(12.0%) | 0.800 (0.037-17.196) | 0.887 | - | - |
| Surgical margin | R0 | 44(95.7%) | 19(76.0%) | 7.250 (2.042-25.736) | **0.002** | 4.857 (0.992-23.787 ) | 0.051 |
|  | R1 | 0(0.0%) | 5(20.0%) | - | - | - | - |

**Supplementary Table 3.** Comparison of clinical trials of local advanced NSCLC

| Trial | Trial type | Patients’ stage | Neoadjuvant treatment mode | R0% (n) | MPR% (n) | pCR% (n) | DFS/PFS/EFS (survival, median/%) | OS% (median/%) | Median follow-up (months) |
| --- | --- | --- | --- | --- | --- | --- | --- | --- | --- |
| Provencio et al, 2020 (1) | prospective, single-arm, phase II | IIIA | Chemoimmunotherapy | 100% (41/41) | 82.9%(34/41) | 63.4% (26/41) | PFS:69.6% (3-year) | 81.9% (3-year) | 38.0 |
| Rothschild et al, 2021 (2) | prospective, single-arm, phase II | IIIA | Chemoimmunotherapy | 93% (51/55) | 62%(34/55) | 18%(10/55) | EFS: 68% (2-year) | 83% (2-year) | 28.6 |
| Sun et al, 2024 (3) | prospective, single-arm, phase II | IIIA/IIIB | Chemoimmunotherapy | 100% (20/20) | 65.0%(13/20) | 40.0%(8/20) | DFS: 75% (2-year) | 80% (2-year) | 39.7 |
| Lei et al, 2023 (4) | prospective, randomized clinical trial, phase II | IIIA/IIIB | Chemoimmunotherapy | - | 65.1%(28/43) | 32.6%(14/43) | DFS: 78.4% (2-year) | - | 14.1 |
|  |  |  | Chemotherapy | - | 15.6%(7/45) | 8.9% (4/45) | DFS: 71.7% (2-year) | - |  |
| Wang et al，2024* | retrospective | IIIA/IIIB | Chemoimmunotherapy | 95.7% (44/46) | 63.0%(29/46) | 34.8%(16/46) | DFS: 85.0% (4-year) | 85.1% (4-year) | 35.0 |
|  |  |  | Chemotherapy | 76.0 (19/25) | 16.0%(4/25) | 8.0%(2/25) | DFS: 21.8% (4-year) | 63.8% (4-year) |  |

R0, R0 resection (no residual tumor); MPR, major pathological response; pCR, pathological complete response; DFS, disease-free survival; PFS, progress-free survival; EFS, event-free survival; OS, overall survival.

*Represents this study.

References:

Provencio M, Nadal E, Insa A, Garcıa-Campelo MR, Casal-Rubio J, Domine M, et al. Neoadjuvant chemotherapy and nivolumab in resectable non-mall-cell lung cancer (NADIM): an open-label, multicenter, single-arm, phase 2 trial. Lancet Oncol. (2020) 21:1413–22. <https://linkinghub.elsevier.com/retrieve/pii/S1470204520304538>.

Rothschild SI, Zippelius A, Eboulet EI, Savic Prince S, Betticher D, Bettini A, Q26 et al. SAKK 16/14: durvalumab in addition to neoadjuvant chemotherapy in patients with stage IIIA(N2) non–small-cell lung cancer—A multicenter single-arm phase II trial. J Clin Oncol. (2021) 39:2872–80. doi: 10.1200/JCO.21.00276

Shu C, Gainor J, Awad M, Chiuzan C, Grigg C, Pabani A, et al. Neoadjuvant atezolizumab and chemotherapy in patients with resectable non-small-cell lung cancer: an open-label, multicenter, single-arm, phase 2 trial. Lancet Oncol. (2020) 21. doi: 10.1016/S1470-2045(20)30140-6

Lei J, Zhao J, Gong L, Ni Y, Zhou Y, Tian F, et al. Neoadjuvant camrelizumab plus platinum-based chemotherapy vs chemotherapy alone for chinese patients with resectable stage IIIA or IIIB (T3N2) non–small cell lung cancer: the TD-FOREKNOW randomized clinical trial. JAMA Oncol. (2023) 9:1348–55. doi: 10.1001/jamaoncol.2023.2751
